# Virtual reality–based assessment of visuo-vestibular integration in vestibular migraine and migraine: static and dynamic visual vertical and rod-and-frame tests

**DOI:** 10.3389/fneur.2025.1710226

**Published:** 2026-01-15

**Authors:** Hanifi Korkmaz, Sibel Çıplak, Rania Alkahtani

**Affiliations:** 1 Medical Health Services and Vocational School, Malatya Turgut Ozal University, Malatya, Türkiye; 2 Department of Neurology, Faculty of Medicine, Malatya Turgut Ozal University, Malatya, Türkiye; 3 Department of Health Communication Sciences, College of Health and Rehabilitation Sciences, Princess Nourah Bint Abdulrahman University, Riyadh, Saudi Arabia

**Keywords:** DHI, dynamic visual vertical, MIDAS, migraine, rod-and-frame, subjective visual vertical, vestibular migraine, virtual reality

## Abstract

**Background:**

Vestibular migraine (VM) is the most common cause of episodic vertigo, yet its diagnostic markers remain limited. Perception of verticality, measured via static subjective visual vertical (SVV), dynamic SVV (DSVV), and the rod-and-frame test (RFT), provides insight into visuo-vestibular integration. Traditional approaches, however, often test these paradigms in isolation with limited ecological validity.

**Objective:**

To evaluate a novel virtual reality (VR)-based protocol combining SVV, DSVV, and RFT to characterize visuo-vestibular integration in VM, migraine without vestibular symptoms (M), and healthy controls (HC).

**Methods:**

Fifty participants (VM *n* = 15, M *n* = 15, HC *n* = 20) completed VR-based SVV, DSVV, and RFT assessments using the BalanceVR system. Disability was assessed using the Dizziness Handicap Inventory (DHI) and Migraine Disability Assessment (MIDAS). Between-group differences were analyzed using Kruskal–Wallis tests with Mann–Whitney U post-hoc comparisons. Correlations between orientation measures and questionnaires were assessed using Spearman’s rho.

**Results:**

VM patients exhibited significantly greater SVV deviations than HC (*p* = 0.015). DSVV errors were elevated in VM vs. HC (*p* = 0.040). RFT showed robust group differences (*p* < 0.001), with the most significant visual dependence in VM. DHI and MIDAS scores were significantly higher in both patient groups than HC (*p* < 0.001). DSVV (*ρ* ≈ 0.45) and RFT (ρ ≈ 0.69) showed stronger correlations with disability than static SVV (ρ ≈ 0.30).

**Conclusion:**

VM is characterized by difficulties in dynamic and visually complex situations, rather than static balance problems. Combining DSVV and RFT with SVV enhances diagnostic sensitivity, explains patients’ daily challenges, and supports rehabilitation approaches aimed at reducing visual dependence and improving sensory balance.

## Introduction

Migraine is an extensive neurological disorder characterized by recurrent headache attacks, frequently accompanied by sensory disturbances such as photophobia, phonophobia, and nausea (1–3). Vestibular migraine (VM), now recognized as one of the most common causes of vertigo. It represents a complex subtype in which migraine symptoms are causally linked with vestibular dysfunction (4, 5). The pathophysiology is believed to involve abnormal multisensory integration and trigeminovascular mechanisms, while the clinical manifestations include unsteadiness, balance problems, and spontaneous or positional vertigo (6).

Subjective visual vertical (SVV), which is operationalized as the perception of verticality, offers a readily available indicator of otolithic and graviceptive processes. In order to test SVV, participants usually align a line to true vertical against a stationary, dark background. By introducing a moving visual background (optokinetic stimuli), Dynamic SVV (DSVV) reveals disruptions in visuo-vestibular integration and enables the quantification of visual dependency (7, 8). Despite research on SVV and DSVV in migraine, the findings are still mixed. Some studies have demonstrated subclinical deviations of SVV in migraineurs (9), whereas others have failed to replicate these findings (10). Meta-analytic evidence nonetheless suggests moderate SVV misperceptions in migraine patients (11). Particularly in VM, SVV deviations seem more stable when standing, indicating compromised neural processes for spatial orientation (12, 13). By highlighting an over-reliance on visual input, dynamic paradigms have been demonstrated to improve diagnostic sensitivity (14).

The rod-and-frame test (RFT) is used to examine how a tilted frame can distort verticality judgments. In practice, it serves as a way to estimate visual dependence. While the test has long been applied in clinical and research settings, more recent Virtual reality (VR)-based versions have shown that it can also capture subtle differences in how people maintain orientation and how strongly they are influenced by surrounding visual cues (15).

This is particularly relevant to VM, where upright perception errors are exacerbated under tilted head or body conditions, indicating disruption of higher-order multisensory processes (13). Despite this growing body of research, significant gaps remain. Most prior work has explored SVV, DSVV, or RFT in isolation, using traditional laboratory-based setups with limited ecological validity. VR systems offer a unique advantage by integrating static, dynamic, and contextual paradigms into a single, standardized environment. Recent work has validated the feasibility of VR-based SVV testing, demonstrating portability and reliability (16). However, no study to date has applied a combined VR protocol incorporating SVV, DSVV, and RFT simultaneously in VM and migraine populations.

The present study aims to address this gap by implementing a VR-based assessment protocol (BalanceVR system) integrating SVV, DSVV, and RFT in patients with vestibular migraine, migraine without vestibular symptoms, and healthy controls. Previous research indicates that vestibular migraine is associated with abnormalities in verticality perception. Static SVV deviations have been reported, although with modest sensitivity (11, 12), while dynamic SVV tasks appear to uncover exaggerated visual dependence and impaired visuo-vestibular integration (8, 14, 17). In addition, tasks that manipulate contextual visual frames, such as the rod-and-frame test, have shown that VM patients are particularly vulnerable to visual reference cues, consistent with higher-order integration deficits (13, 15). On this basis, we hypothesized that (1) patients with vestibular migraine would exhibit greater deviations in static SVV than both migraine and healthy controls, reflecting otolithic dysfunction; (2) both vestibular migraine and migraine patients would display larger errors in DSVV compared with controls, indicating altered visuo-vestibular integration; and (3) vestibular migraine patients would demonstrate heightened visual dependence on the RFT relative to both other groups, consistent with disturbances in multisensory integration. By testing these hypotheses within a unified VR protocol, our study aims to provide a comprehensive and ecologically valid assessment of spatial orientation impairments across the migraine spectrum.

## Methods

### Participants

Three groups were enrolled: (i) migraine (M), diagnosed according to the International Classification of Headache Disorders, 3rd edition (ICHD-3) (3, 18), (ii) vestibular migraine (VM), diagnosed following the Bárány Society and International Headache Society consensus criteria (5), and (iii) healthy controls (HC) without a history of vestibular, neurological, or otological disorders.

Inclusion criteria were age 18–60 years with normal or corrected-to-normal vision. Exclusion criteria included other vestibular disorders (e.g., Ménière’s disease, benign paroxysmal positional vertigo, vestibular neuritis), psychiatric or neurological comorbidities, uncorrected visual impairment, or current use of vestibulotoxic medications. In addition to the predefined exclusion criteria, all participants underwent a comprehensive bedside audiovestibular screening performed by a doctoral-level audiologist (PhD) prior to enrolment. This assessment followed established clinical frameworks for bedside vestibular evaluation (19, 20) and included a detailed battery of vestibulo-ocular and vestibulospinal tests to exclude concomitant peripheral or central vestibular dysfunction. The vestibulo-ocular component comprised bedside head impulse testing, gaze-evoked and endpoint nystagmus assessment, positional maneuvers for benign paroxysmal positional vertigo (Dix–Hallpike and supine roll tests), and head-shaking nystagmus testing. Vestibulospinal function was evaluated using Romberg, tandem Romberg, and Fukuda stepping tests to detect postural asymmetry or directional drift indicative of vestibular impairment. Participants demonstrating any abnormality during these tests, or reporting symptoms suggestive of Ménière’s disease, vestibular neuritis, recurrent BPPV, or other vestibular disorders, were excluded. This systematic screening ensured that the final sample did not contain individuals with concurrent vestibular dysfunction that could confound SVV, DSVV, or RFT outcomes.

Participants in the migraine and vestibular migraine groups were recruited consecutively from the Neurology outpatient clinic of Malatya Training and Research Hospital. Eligible individuals who met the diagnostic criteria (3, 5) were identified by the attending neurologist and referred for study screening. Healthy controls were recruited through institutional announcements among hospital staff and community volunteers and were required to have no history of migraine, vestibular disorders, or neurological disease. All participants were evaluated during interictal periods. Participants who were using or had recently used prophylactic migraine medications (e.g., beta-blockers, topiramate, valproate, calcium-channel blockers, tricyclic antidepressants) were excluded unless they had never initiated prophylaxis or had discontinued treatment at least 4 weeks prior to testing to allow sufficient wash-out and avoid medication-related confounding effects.

The distinction between migraine and vestibular migraine was made using established diagnostic frameworks. Migraine was diagnosed according to the International Classification of Headache Disorders (ICHD-3) criteria, which define migraine based on stereotyped headache characteristics and associated symptoms (21). Vestibular migraine was diagnosed following the Bárány Society and International Headache Society consensus criteria, requiring a history of migraine and at least five episodes of vestibular symptoms of moderate or severe intensity that occur in temporal association with migraine features, after excluding other vestibular disorders (5). Individuals who met migraine criteria but did not report vestibular episodes meeting these criteria were classified into the migraine (M) group. Prior literature supports this distinction, as migrainous vertigo presents a distinct clinical entity with an estimated lifetime prevalence of approximately 1% and requires careful differential diagnosis to avoid misclassification (21, 22).

#### Clinical questionnaires

Self-perceived disability was assessed with two validated instruments:

- *Dizziness Handicap Inventory (DHI):* The DHI consists of 25 items across physical, functional, and emotional domains, with scores ranging from 0 to 100 and categorized as mild (0–30), moderate (31–60), or severe (61–100). It is a widely validated tool for assessing vestibular-related disability (23–25). The Turkish validated version was used (26).
- *Migraine Disability Assessment (MIDAS):* Migraine-related disability was evaluated using the MIDAS questionnaire, a widely validated instrument developed to quantify the functional impact of migraine over a 3-month period (27). MIDAS includes five core items that assess the number of days in which migraine resulted in: (1) missed work or school, (2) ≥ 50% reduced productivity at work or school, (3) missed household responsibilities, (4) ≥ 50% reduced productivity in household tasks, and (5) missed social, family, or leisure activities. The total MIDAS score is computed by summing these five items and is categorized into four internationally accepted disability grades: Grade I (0–5, little or no disability), Grade II (6–10, mild disability), Grade III (11–20, moderate disability), and Grade IV (≥21, severe disability). The MIDAS grading system provides a standardized and clinically meaningful evaluation of migraine burden and is widely used in both research and clinical practice (28). The Turkish validated version was applied in this study (29).

#### Apparatus and procedures

Testing was delivered via the Virtualis Balance VR system (Virtualis, France), an immersive VR platform designed to assess visuo-vestibular orientation. The system standardizes stimulus presentation, minimizes extraneous spatial cues, and allows integration of static SVV, dynamic SVV, and rod-and-frame paradigms within a controlled environment. Prior work has established the feasibility and reliability of VR-based SVV/DSVV procedures, demonstrating strong agreement with conventional bucket and dome methods while enhancing ecological validity and portability (8, 16). In this study, the term *ecological validity* refers to the capacity of the VR environment to approximate real-world visuo-vestibular conditions by providing immersive, naturalistic visual surroundings and controlled sensory conflict. Beyond assessment, such VR-based systems have been increasingly applied in the evaluation and rehabilitation of patients with migraine, vestibular migraine, peripheral vestibulopathies, and persistent postural-perceptual dizziness (PPPD), where dynamic sensory reweighting and exposure to structured visual environments are clinically relevant (30). Detailed module parameters in this study followed the manufacturer’s specifications (31–33).

Participants were seated in a dimly lit, quiet room with their feet unsupported to minimize proprioceptive input and reduce somatosensory influence on verticality judgments (34). All SVV tasks, including the DSVV, were performed in this seated position. In the dynamic condition, an optokinetic background was presented through the head-mounted display, and participants adjusted the luminous line using a joystick until they perceived it as vertical. This ensured controlled visual motion stimulation while preventing external spatial cues.

##### Static subjective visual vertical

**Figure 1:**
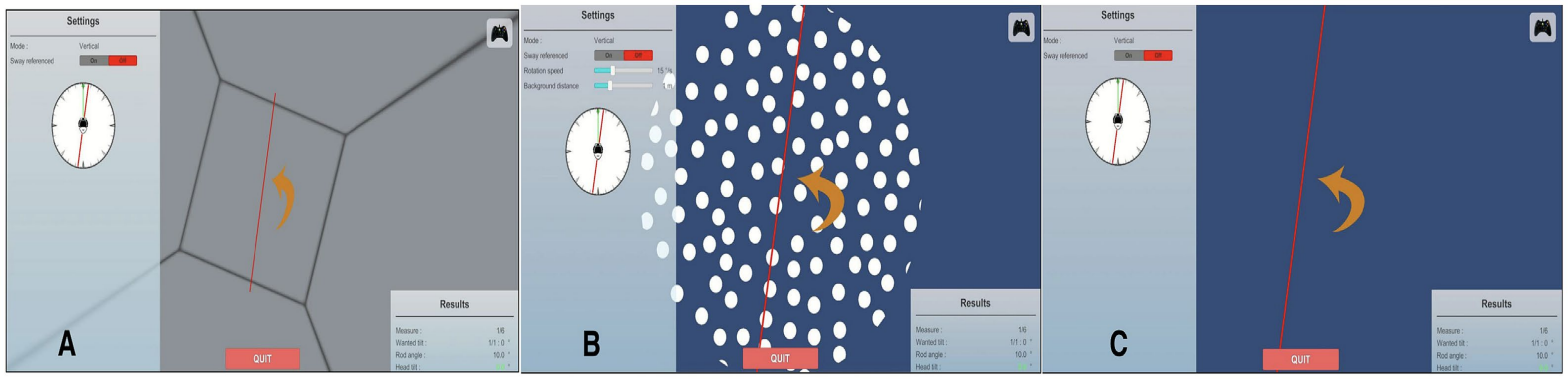
Representative screenshots of the BalanceVR test modules: **(A)** Rod and frame test (RFT). **(B)** Dynamic subjective visual vertical (DSVV). **(C)** Static subjective visual vertical (SVV).

The static SVV task was carried out with the BalanceVR system (Figure 1A). Participants sat in a dim room, wearing a head-mounted display, and observed a single red luminous line against a completely dark, uniform background (Figure 1C). The absence of external spatial references forced judgments of verticality to rely on vestibular otolith input (14, 35). At the start of each trial, the red line appeared at one of several randomized tilts (±5°, ±10°, ±15°, ±20°, ±40°). Using an Xbox joystick, participants rotated the line until they believed it was aligned with gravity. Each participant completed 10 trials. Deviation was defined as the angular distance between the adjusted line and 0°, with clockwise responses coded as positive and counterclockwise as negative. Compared with dome or bucket setups, the VR system allowed more precise stimulus control and reduced reliance on proprioceptive or visual cues. The VR-SVV module incorporates built-in factory calibration to ensure accurate representation of gravitational vertical. Although no separate in-study calibration against a bucket test was conducted, prior validation studies have shown strong concordance between VR-based SVV measures and traditional bucket and dome paradigms (14, 16).

##### Dynamic subjective visual vertical

In the DSVV condition, the background consisted of moving white dots displayed on a blue surface, producing an optokinetic effect (Figure 1B). As in the static task, there were no linear features or borders to provide reference cues. The same red line was presented at randomized starting tilts (±5°, ±10°, ±15°, ±20°, ±40°). Participants rotated the line with the joystick until it appeared aligned with gravity. Deviations were recorded as positive for clockwise (CW) and negative for counterclockwise (CCW). Background rotation speed was fixed at 30°/s, and the direction depended on the starting angle: CW if the tilt was positive and CCW if negative. Stimuli were evenly distributed across both directions (16).

##### Rod-and-frame test

For the RFT, participants were immersed in a tilted rectangular virtual box that acted as the misleading frame (Figure 1A). The frame was angled at ±10°, ±20°, or ±30° relative to true vertical. Within this environment, a red luminous line appeared in different randomized orientations across 10 trials. Participants used the joystick to rotate the line until they judged it to be vertical in real space, ignoring the tilted frame. Deviations from the true vertical indicated the degree of visual dependence. This VR-based version expands the traditional rod-and-frame paradigm by embedding the participant in a three-dimensional tilted context, increasing ecological validity and immersion while minimizing external spatial cues (15). Additional implementation details are available in the module tutorials (31–33). Conceptually, the RFT measures visual dependence, the degree to which orientation judgments rely on external visual cues rather than graviceptive input. When the surrounding frame is tilted, individuals with higher visual dependence systematically deviate their rod adjustments toward the frame orientation (36). Clinically, increased frame dependency has been reported in patients with vestibular disorders, supporting its diagnostic utility (37). Scoring is based on the angular deviation from true vertical, with larger deviations indicating stronger visual influence. Unlike SVV or DSVV, which primarily assess otolith–visual integration under minimal or moving visual backgrounds, the RFT engages higher-order contextual processing and conflict resolution, offering enhanced sensitivity for detecting sensory reweighting abnormalities such as those observed in vestibular migraine.

#### Normalization and reference values

HCs were assessed first to establish normative data. Results showed that SVV deviations remained within ±2°, and DSVV within 1–3°, consistent with prior reports (14, 16, 35). Off-axis tilts did not exceed 11° in healthy subjects, confirming alignment with established thresholds (38). These values validated that the VR-based paradigm reproduces traditional results while enhancing ecological validity through immersive presentation.

### Statistical analysis

All analyses were performed in IBM SPSS v22. Distributional assumptions were assessed with Shapiro–Wilk and Q–Q plots within each group. Given *n* < 30 per group and potential outliers, non-parametric tests were prespecified. Between-group differences were evaluated with Kruskal–Wallis; when significant, adjusted pairwise Mann–Whitney U tests were planned. Within-group SVV vs. DSVV comparisons used Wilcoxon signed-rank tests. Associations among orientation measures and questionnaire scores were examined using Spearman’s rank correlation. Two-tailed *α* = 0.05. Sample sizes are comparable to prior studies reporting significant group effects with similar paradigms (14, 39).

In addition to test statistics and *p*-values, effect sizes were calculated for all nonparametric analyses. For Kruskal–Wallis tests, eta-squared [η^2^ = H/(N – 1)] was computed to quantify the proportion of variance explained. For Mann–Whitney U tests, effect size r was calculated as *r* = Z/√N, representing the standardized magnitude of group differences. For Wilcoxon signed-rank tests, effect size r (Z/√N) was also reported. Spearman’s rho coefficients inherently represent effect size for correlational analyses. For post-hoc pairwise Mann–Whitney U comparisons, Bonferroni correction was applied to adjust the significance threshold (αadj = 0.05/number of comparisons) to control for Type I error. To complement the nonparametric *p*-values, effect sizes (η^2^ and r) were examined as indicators of statistical precision, particularly for assessing non-significant contrasts such as VM–M, where small effect sizes may reflect limited statistical power.

### Ethical consideration

The study was conducted in accordance with the principles of the Declaration of Helsinki and was approved by the Institutional Ethics Committee of Malatya Turgut Özal University (approval number: 2025/256). Written informed consent was obtained from all participants.

## Results

Demographic characteristics of the study participants are presented in Table 1. The total sample consisted of 50 individuals. Participants were relatively evenly distributed across age groups, with balance representation of sex.

**Table 1:** Demographic characteristics of participants.

Variable	Category	*N*	%
Age	19–25	9	18.0
	26–35	11	22.0
	36–45	11	22.0
	46–55	12	24.0
	56–59	7	14.0
	Total	50	100
Sex	Female	25	50.0
	Male	25	50.0
	Total	50	100

Values are presented as frequency (N) and percentage (%).

As shown in Table 2, static SVV differed significantly across groups (*H* = 8.367, *p* = 0.015). Pairwise Mann–Whitney U tests indicated that patients with vestibular migraine (VM; mean rank = 33.23) exhibited greater deviations compared with healthy controls (HC; mean rank = 18.93). No significant differences were observed between the migraine group (M; mean rank = 26.53) and the other groups.

**Table 2:** Group comparisons of VR-based orientation measures (SVV, DSVV, RFT) and questionnaire outcomes (DHI, MIDAS) among groups.

Measure	M (*N* = 15) mean rank	VM (*N* = 15) mean rank	HC (*N* = 20) mean rank	H (DF = 2)	*P-*value	Effect size (η^2^)	*Post hoc* comparisons
SVV (°)	26.53	33.23	18.93	8.367	0.015*	0.17	VM-HC
DSVV (°)	25.73	32.60	20.00	6.416	0.040*	0.13	VM-HC
RFT (°)	29.47	40.47	11.30	35.916	<0.001*	0.73	M-VM, M-HC, VM-HC
DHI score	30.30	40.20	10.88	37.069	<0.001*	0.76	M-VM, M-HC, VM-HC
MIDAS score	36.03	34.67	10.73	34.450	<0.001*	0.70	M-HC, VM-HC

Values are presented as mean ranks. Group comparisons were performed using the Kruskal–Wallis test (H = test statistic, df = 2). *p < 0.05 indicates statistical significance. Post-hoc comparisons column will be completed based on further pairwise analyses. M, Migraine; VM, Vestibular Migraine; HC, Healthy Controls; SVV, Subjective Visual Vertical; DSVV, Dynamic Subjective Visual Vertical; RFT, Rod-and-Frame Test; DHI, Dizziness Handicap Inventory; MIDAS, Migraine Disability Assessment. Significant post-hoc comparisons reflect results after Bonferroni correction.

For DSVV, a significant group effect was also found (*H* = 6.416, *p* = 0.040). Post-hoc analyses revealed that VM patients (mean rank = 32.60) showed higher deviations than HC participants (mean rank = 20.00), whereas differences involving the migraine group (M; mean rank = 25.73) did not reach statistical significance.

In the RFT, robust group differences emerged (*H* = 35.916, *p* < 0.001). Both migraine (M; mean rank = 29.47) and vestibular migraine (VM; mean rank = 40.47) patients demonstrated greater deviations than healthy controls (HC; mean rank = 11.30), and VM patients also scored higher than migraine patients, indicating the most substantial impairment in the VM group.

Regarding self-reported outcomes, DHI scores were significantly elevated in patient groups (*H* = 37.069, *p* < 0.001). Both migraine (M; mean rank = 30.30) and vestibular migraine (VM; mean rank = 40.20) groups scored higher than HC (mean rank = 10.88), with VM patients showing the greatest handicap levels. Similarly, MIDAS scores revealed significant group differences (*H* = 34.450, *p* < 0.001). Both migraine (M; mean rank = 36.03) and VM (mean rank = 34.67) patients scored higher than HC (mean rank = 10.73), reflecting a greater headache-related disability in the patient groups. Corresponding effect sizes (r) for pairwise comparisons were also calculated to reflect the magnitude of group differences. Between-group comparisons for SVV, DSVV, and RFT are summarized in Table 2. Descriptive statistics (median and interquartile range; IQR) for all measures are presented in Table 3.

**Table 3:** Descriptive statistics (median and IQR) for VR-based orientation measures and questionnaire scores across groups.

Measure	Group	*N*	Median [IQR]
SVV (°)	M	15	0.57 [0.20–1.88]
SVV (°)	VM	18	1.58 [0.49–2.28]
SVV (°)	HC	20	0.18 [−0.43–0.72]
DSVV (°)	M	13	0.85 [−0.11–2.08]
DSVV (°)	VM	15	3.22 [−0.61–5.14]
DSVV (°)	HC	20	0.47 [−1.24–1.51]
RFT (°)	M	15	6.31 [5.84–7.15]
RFT (°)	VM	18	10.31 [8.31–15.64]
RFT (°)	HC	20	3.30 [3.01–3.94]
DHI (score)	M	15	22.0 [19.0–29.5]
DHI (score)	VM	18	47.5 [40.5–51.0]
DHI (score)	HC	20	10.5 [7.75–12.75]
MIDAS (score)	M	15	13.0 [6.5–21.0]
MIDAS (score)	VM	18	9.5 [8.25–14.0]
MIDAS (score)	HC	20	2.0 [1.0–3.25]

Values represent median and interquartile range (IQR). Descriptive statistics were calculated to complement the nonparametric group comparisons presented in Table 2 and to improve the clinical interpretability of VR-based orientation measures and questionnaire scores. M, Migraine; VM, Vestibular Migraine; HC, Healthy Controls.

Within-group comparisons of static and dynamic SVV are summarized in Table 4. Wilcoxon Signed-Rank tests revealed no significant differences between SVV and DSVV in any of the groups. Specifically, migraine patients (*Z* = −0.170, *p* = 0.865), vestibular migraine patients (*Z* = −1.079, *p* = 0.281), and healthy controls (*Z* = 0.000, *p* = 1.000) all demonstrated comparable performance on static and dynamic verticality perception tasks, indicating that the addition of optokinetic stimulation did not significantly alter verticality estimates within groups. Mean-rank ordering (VM > M > HC for RFT; VM > HC for SVV/DSVV) indicates progressively greater visual context susceptibility and motion-related bias in patient groups, without asserting pairwise directionality beyond the reported *post-hoc* results.

**Table 4:** Within-group comparisons of static SVV and DSVV (Wilcoxon signed-rank test).

Group	*N*	*Z*	Effect size (r)	*P*-value
M	15	−0.170	0.04	0.865
VM	15	−1.079	0.28	0.281
HC	20	0.000	0.00	1.000

Wilcoxon Signed-Rank Test was used to compare static SVV and dynamic SVV within each group. No significant differences were found (all *p* > 0.05). M, Migraine; VM, Vestibular Migraine; HC, Healthy Controls; SVV, Subjective Visual Vertical; DSVV, Dynamic Subjective Visual Vertical.

**Figure 2:**
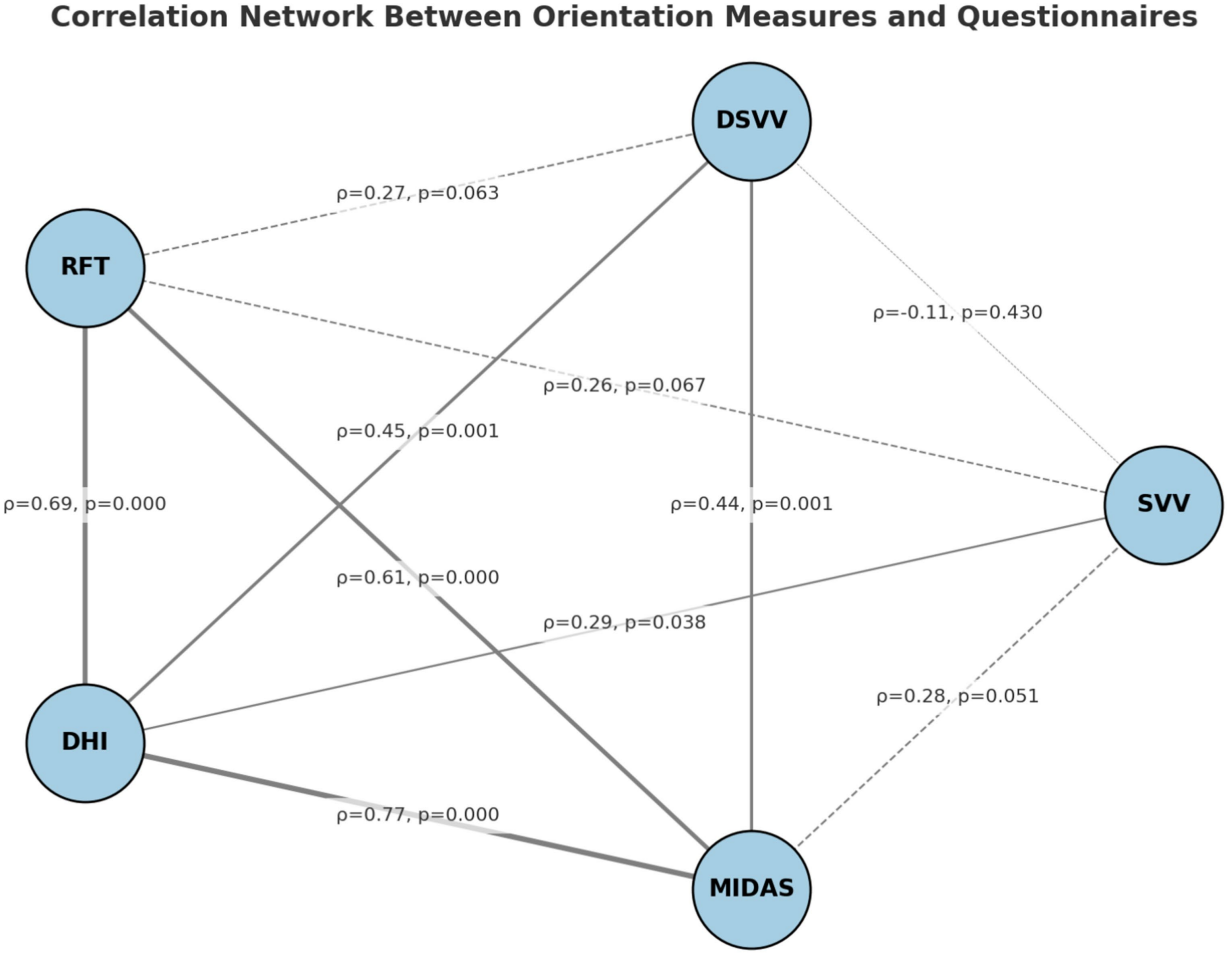
Correlation network between orientation measures (SVV, DSVV, RFT) and questionnaire scores (DHI, MIDAS). Values represent Spearman’s rho (*ρ*) with corresponding *p*-values. Line thickness reflects correlation strength. Bold lines indicate statistically significant associations (*p* < 0.05).

As shown in Table 5, Spearman’s correlation analysis revealed several significant associations. Static SVV scores were modestly correlated with DHI (*ρ* = 0.30, *p* = 0.038), whereas their association with MIDAS did not reach statistical significance (ρ = 0.28, *p* = 0.051). Dynamic SVV showed stronger correlations with both DHI (ρ = 0.45, *p* = 0.001) and MIDAS (ρ = 0.44, *p* = 0.001). The RFT exhibited robust positive associations with DHI (ρ = 0.69, *p* < 0.001) and MIDAS (ρ = 0.61, *p* < 0.001). Moreover, the two questionnaire measures (DHI and MIDAS) were strongly correlated with each other (ρ = 0.77, *p* < 0.001). These findings indicate that greater perceptual deviations are consistently linked with higher levels of self-reported disability and migraine-related burden. Correlation coefficients and significance levels are reported in Table 5, while Figure 2 provides a visual representation of the correlation network. Presenting both formats allows for precise statistical interpretation as well as intuitive visualization of the overall association pattern.

**Table 5:** Correlations between VR-based orientation measures (SVV, DSVV, RFT) and questionnaire outcomes (DHI, MIDAS).

Variables	SVV (°)	DSVV (°)	RFT (°)	DHI (score)	MIDAS (score)
SVV (°)	1.00	ρ = −0.11, *p* = 0.430	ρ = 0.26, *p* = 0.067	ρ = 0.30, *p* = 0.038*	ρ = 0.28, *p* = 0.051
DSVV (°)		1.00	ρ = 0.27, *p* = 0.063	ρ = 0.45, *p* = 0.001**	ρ = 0.44, *p* = 0.001**
RFT (°)			1.00	ρ = 0.69, *p* < 0.001**	ρ = 0.61, *p* < 0.001**
DHI				1.00	ρ = 0.77, *p* < 0.001**
MIDAS					1.00

SVV, Subjective Visual Vertical; DSVV, Dynamic Subjective Visual Vertical; RFT, Rod-and-Frame Test; DHI, Dizziness Handicap Inventory; MIDAS, Migraine Disability Assessment. *p < 0.05 (two-tailed). *p < 0.01 (two-tailed). Correlation coefficients (ρ) are Spearman’s rho values.

## Discussion

This study is the first to apply a comprehensive virtual reality (VR) protocol combining static subjective visual vertical (SVV), dynamic subjective visual vertical (DSVV), and the Rod-and-Frame Test (RFT) to interrogate visuo-vestibular integration across vestibular migraine (VM), migraine without vestibular symptoms, and healthy controls. In our cohort, group differences were present for all three orientation measures, with the largest separation observed for RFT. Concordant elevations in DHI and MIDAS further indicated that perceptual deviations co-varied with clinical burden. Taken together, these data emphasize that dynamic and context-dependent tasks capture clinically meaningful vulnerability beyond that revealed by static SVV alone.

Placed against prior work, our SVV findings align with reports that upright SVV can distinguish VM from controls but with limited diagnostic accuracy. Li et al. (12) showed modest sensitivity and specificity for upright SVV in VM, a pattern mirrored here by a significant VM–control difference but no reliable separation between VM and migraine. Similarly, Bogle et al. (40) found abnormal static SVV tilt or variance in a majority of VM patients, supporting utriculo-ocular pathway involvement yet also underscoring that static measures alone incompletely characterize dysfunction. By contrast, dynamic paradigms were more informative. Miller and Crane (14) demonstrated that visual motion magnifies verticality error in migraine and VM, consistent with impaired sensory reweighting; Ashish et al. (39) likewise reported dynamic SVV/SVH abnormalities during interictal periods. Our DSVV results reproduce this pattern: group effects emerged and DSVV correlated more strongly than SVV with disability indices, indicating greater clinical salience of dynamic cues. The clinical salience of dynamic and contextual measures was reinforced by their stronger correlations with DHI and MIDAS relative to static SVV. This pattern suggests that visually driven bias (DSVV) and frame dependence (RFT) better track day-to-day symptom burden than tonic otolith bias alone, converging with reports that visual motion disproportionately perturbs verticality judgments in migraine/VM (14) and that dynamic SVV abnormalities persist interictally in VM (39). Finally, our VR-based RFT shows pronounced visual dependence in VM, extending observations that upright perception errors are exacerbated by altered frames of reference in VM (13). The present RFT results therefore provide convergent evidence for heightened reliance on visual context in VM.

Interpreted in light of our a-priori hypotheses, the pattern is internally consistent. Hypothesis 1 posited greater SVV deviation in VM than in migraine and controls. Our data support Hypothesis 1 relative to controls, but not conclusively versus migraine; thus, Hypothesis 1 is partially supported. Hypothesis 2 predicted that both VM and migraine would exhibit larger DSVV errors than controls. We observed a robust VM–control difference whereas the migraine–control contrast did not reach significance; Hypothesis 2 is therefore partially supported. Hypothesis 3 anticipated heightened visual dependence in VM as indexed by RFT; here, VM exceeded migraine, and both exceeded controls, providing clear support for Hypothesis 3. The correlation structure is coherent with this hierarchy: RFT and DSVV showed moderate-to-strong associations with DHI and MIDAS, whereas SVV displayed weaker or borderline relations, suggesting that dynamic/contextual demands map more closely onto patient-reported disability (12–14, 39, 40). Given the modest sample sizes, the study may have been underpowered to detect subtle differences between vestibular migraine and migraine groups, a limitation consistent with the small effect sizes observed for these contrasts.

Several converging factors may explain the lack of significant differences between vestibular migraine and migraine groups in SVV and DSVV. First, both groups may share overlapping cortical hyperexcitability and altered sensory gain mechanisms associated with migraine, leading to similar subclinical visuo-vestibular abnormalities in the absence of overt vestibular signs. Second, the modest sample size (*n* = 15 per group) may limit statistical power to detect subtle effects even when medium effect sizes are present. Third, because testing was performed during interictal periods, VM-specific abnormalities may have partially normalized outside of acute attacks. Finally, SVV and DSVV primarily probe lower-level otolithic and visuo-vestibular interactions, whereas the Rod-and-Frame Test reflects higher-order context-dependent integration, which may explain why RFT more effectively differentiated VM from migraine.

Mechanistically, the selective amplification of error by visual motion (DSVV) and by tilted frames (RFT) points beyond a purely peripheral otolith lesion. While upright SVV abnormalities may index utriculo-ocular pathway involvement (12, 40), the present hierarchy—RFT ≥ DSVV > SVV—aligns with a higher-order multisensory integration disturbance in VM. This view is consistent with migraine as a disorder of sensory gain control and visuo-vestibular reweighting (41) and with experimental evidence that moving visual stimuli disproportionately bias vertical perception in migraine/VM (14).

Clinically, these findings argue for incorporating DSVV and RFT—alongside SVV—into VM workups to refine phenotyping of visual motion sensitivity and frame dependence (14, 39). Counseling can address visually busy environments and structured visual frames that bias orientation, with graded exposure strategies to mitigate exacerbations. The VR framework also provides a pathway for rehabilitation: progressively dosing visual motion and frame tilt to train sensory reweighting and reduce visual dependence, consistent with adaptation/habituation principles and prior VR feasibility data (15, 16). Tracking DSVV/RFT alongside DHI/MIDAS offers pragmatic outcome markers, as reductions in dynamic or frame-dependent error should parallel improvements in perceived handicap.

Beyond mechanistic inference, these findings carry practical implications for clinical workflows. Because dynamic and frame-based deviations proved more sensitive than static SVV, diagnostic workups for VM should incorporate DSVV and RFT alongside SVV to improve phenotyping of visual dependence and motion sensitivity (14, 39). Patient counseling can explicitly address visually busy environments and structured frames that bias orientation, guiding environmental pacing and trigger management. The VR framework used here also suggests a rehabilitation path: graded exposure to visual motion and tilted frames to train sensory reweighting and reduce visual dependence—an extension of adaptation/habituation principles that VR can standardize and dose (15, 16). Finally, pairing DSVV/RFT metrics with DHI/MIDAS may provide responsive outcome markers, as decreases in dynamic or frame-dependent error should parallel reductions in perceived handicap.

### Strengths and limitations

This work has notable strengths: a VR-based, integrative protocol that unifies static, dynamic, and contextual verticality tasks within a cue-controlled environment; and the linkage of perceptual metrics to validated disability indices, bridging laboratory measures to patient experience. Limitations include a modest sample size that precluded detailed subgroup stratification (e.g., aura), and a cross-sectional design that cannot resolve ictal–interictal dynamics or treatment responsiveness. Nevertheless, our sample sizes are comparable to prior studies reporting group effects in similar paradigms (14, 39). Future studies should replicate these findings in larger cohorts and across ictal versus interictal phases. Integrating VR paradigms with task-based Functional Magnetic Resonance Imaging (fMRI) or electroencephalogram (EEG) could help localize visuo-vestibular integration.

## Conclusion

In conclusion, a VR-based battery integrating SVV, DSVV, and RFT revealed that VM is characterized less by static otolith bias and more by dynamic/contextual susceptibility and visual dependence. Static SVV differentiated VM from controls but only partially supported a VM–migraine separation, whereas DSVV and especially RFT showed clearer group stratification and stronger ties to disability. These findings partially support Hypothesis 1 and Hypothesis 2, fully support Hypothesis 3, and collectively argue that dynamic and frame-based assessments offer superior clinical relevance for diagnosis, monitoring, and rehabilitation planning in VM.

## Data Availability

The original contributions presented in the study are included in the article/Supplementary material, further inquiries can be directed to the corresponding author.
