# Supplementary material for: Virtual reality–based assessment of visuo-vestibular integration in vestibular migraine and migraine: static and dynamic visual vertical and rod-and-frame tests
Source: Front Neurol. 2026 Jan 15;16:1710226. doi: 10.3389/fneur.2025.1710226 (PMC12852887; doi:10.3389/fneur.2025.1710226)
Supplement: Supplementary file 1 [file Data_Sheet_1.pdf]

Patient: Participant (de-identified)

Age:Between 18-40

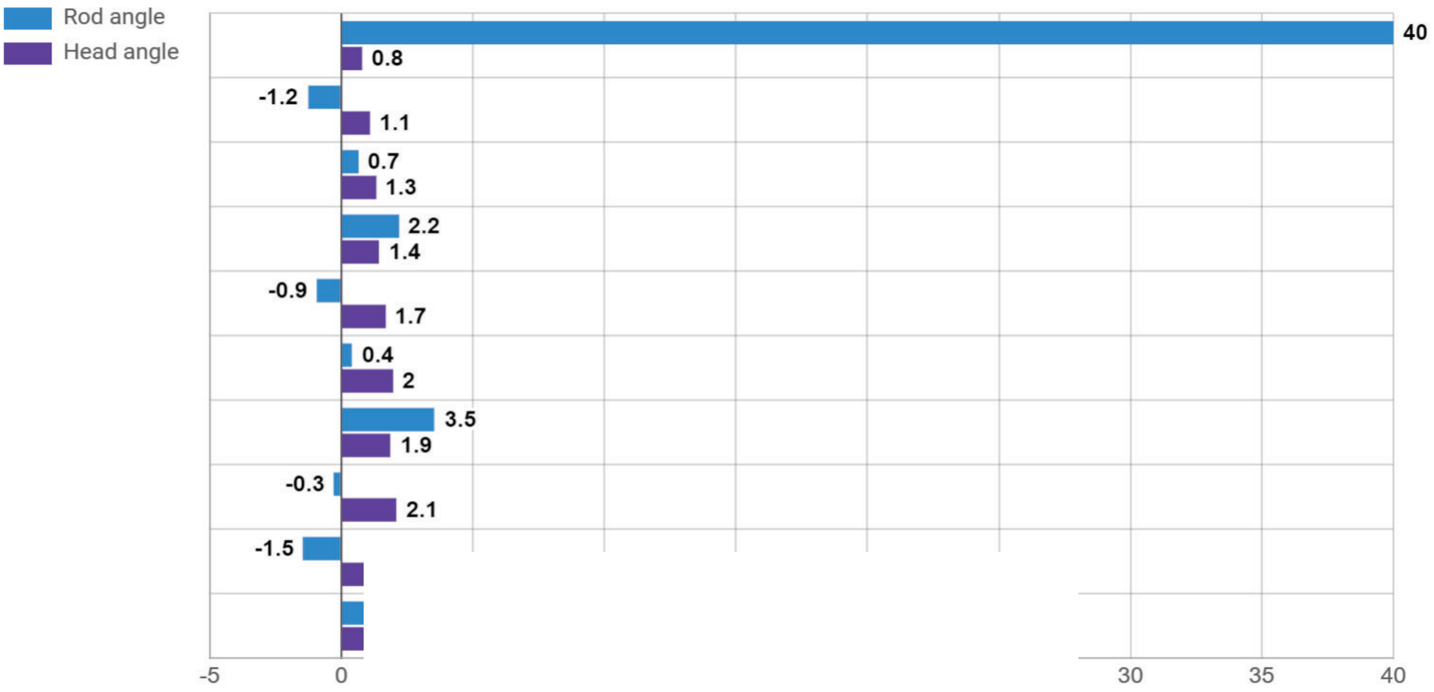

Copyright 2025. Virtualis, all rights reserved

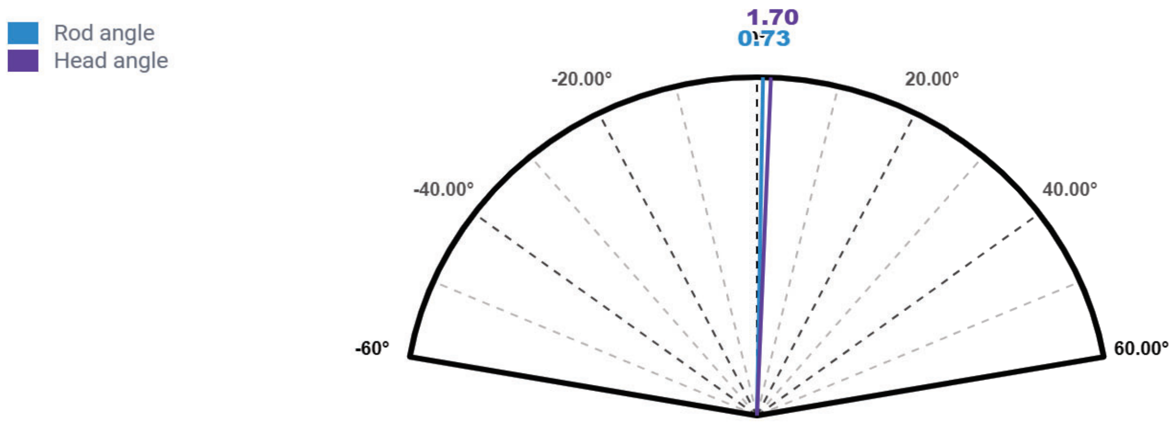

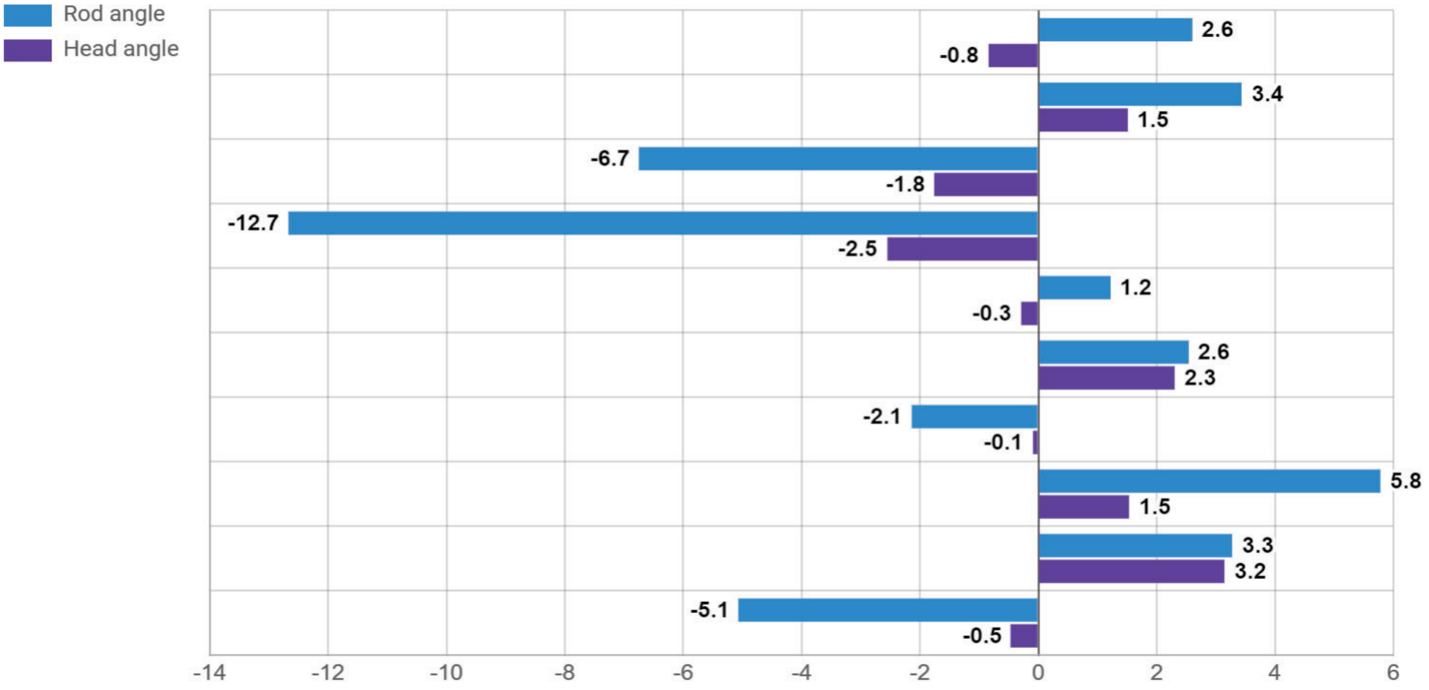

Copyright 2025. Virtualis, all rights reserved

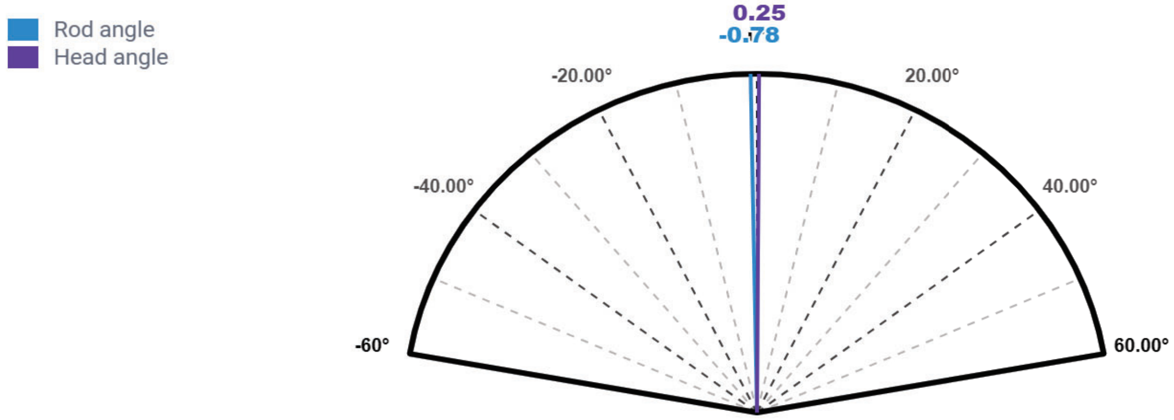

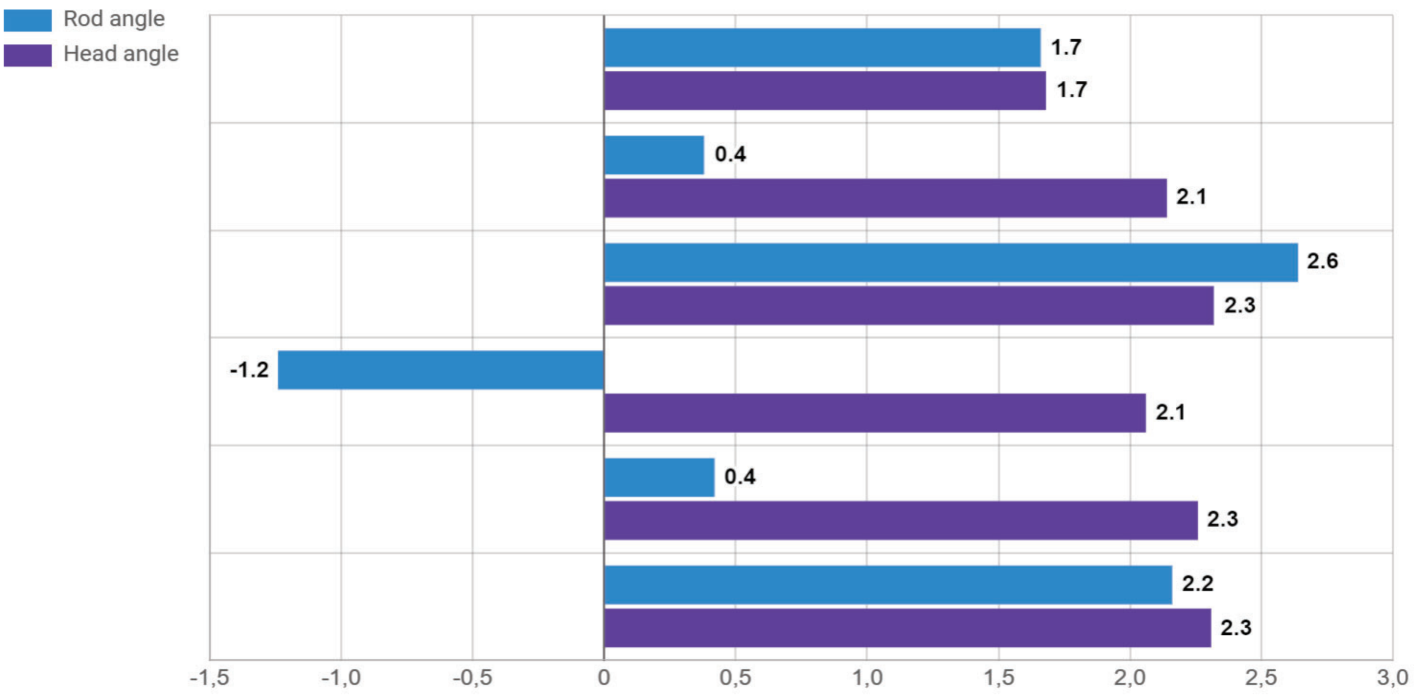

Copyright 2025. Virtualis, all rights reserved

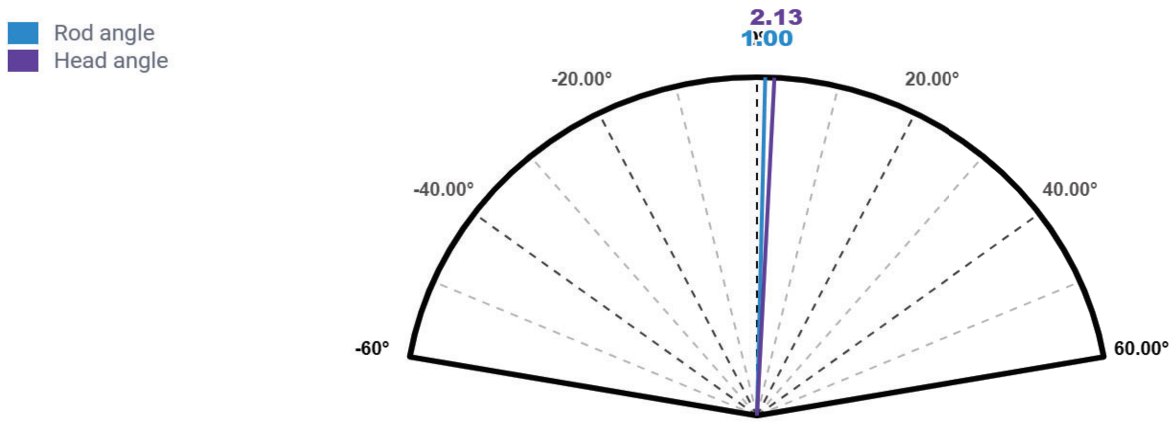

Copyright 2025. Virtualis, all rights reserved
